# *LAMA2* and *LOXL4* are candidate FSGS genes

**DOI:** 10.1186/s12882-021-02524-6

**Published:** 2021-09-26

**Authors:** Poornima Vijayan, Saidah Hack, Tony Yao, Mohammad Azfar Qureshi, Andrew D. Paterson, Rohan John, Bernard Davenport, Rachel Lennon, York Pei, Moumita Barua

**Affiliations:** 1 Department of Molecular Genetics, University of Toronto, Toronto, Canada; 2 Division of Nephrology, University Health Network, Toronto, Canada; 3 Toronto General Hospital Research Institute, Toronto General Hospital, Toronto, Canada; 4 Division of Epidemiology and Biostatistics, Dalla Lana School of Public Health, Toronto, Canada; 5 Institute of Medical Sciences, University of Toronto, Toronto, Canada; 6 Program in Genetics and Genomic Biology, The Hospital for Sick Children, Toronto, Canada; 7 Department of Laboratory Medicine and Pathology, Toronto General Hospital, Toronto, Canada; 8 Wellcome Centre for Cell-Matrix Research, Division of Cell-Matrix Biology and Regenerative Medicine, School of Biological Sciences, Faculty of Biology Medicine and Health, The University of Manchester, Manchester Academic Health Science Centre, Manchester, UK

**Keywords:** Hereditary FSGS, Basement membrane, *LAMA2*, *LOXL4*

## Abstract

**Background:**

Focal and segmental glomerulosclerosis (FSGS) is a histologic pattern of injury that characterizes a wide spectrum of diseases. Many genetic causes have been identified in FSGS but even in families with comprehensive testing, a significant proportion remain unexplained.

**Methods:**

In a family with adult-onset autosomal dominant FSGS, linkage analysis was performed in 11 family members followed by whole exome sequencing (WES) in 3 affected relatives to identify candidate genes.

**Results:**

Pathogenic variants in known nephropathy genes were excluded. Subsequently, linkage analysis was performed and narrowed the disease gene(s) to within 3% of the genome. WES identified 5 heterozygous rare variants, which were sequenced in 11 relatives where DNA was available. Two of these variants, in *LAMA2* and *LOXL4*, remained as candidates after segregation analysis and encode extracellular matrix proteins of the glomerulus. Renal biopsies showed classic segmental sclerosis/hyalinosis lesion on a background of mild mesangial hypercellularity. Examination of basement membranes with electron microscopy showed regions of dense mesangial matrix in one individual and wider glomerular basement membrane (GBM) thickness in two individuals compared to historic control averages.

**Conclusions:**

Based on our findings, we postulate that the additive effect of digenic inheritance of heterozygous variants in *LAMA2* and *LOXL4* leads to adult-onset FSGS. Limitations to our study includes the absence of functional characterization to support pathogenicity. Alternatively, identification of additional FSGS cases with suspected deleterious variants in *LAMA2* and *LOXL4* will provide more evidence for disease causality. Thus, our report will be of benefit to the renal community as sequencing in renal disease becomes more widespread.

**Supplementary Information:**

The online version contains supplementary material available at 10.1186/s12882-021-02524-6.

## Background

Focal and segmental glomerulosclerosis (FSGS) is a histologic lesion with varied causes which include putative circulating factor(s), monogenic etiologies and hyperfiltering conditions [1, 2]. Monogenic FSGS has extensive genetic heterogeneity with 59 implicated genes and the list continues to expand, facilitated by the adoption of next-generation sequencing technologies [3].

Defects in basement membrane proteins of the kidney such as those encoded by type IV collagen and LAMβ2 are amongst the monogenic causes of FSGS [1, 2]. Through broader sequencing efforts, reports over the past several years have identified an under-recognition of pathogenic variants in type IV collagen, causative for Alport syndrome, in patients presenting with a range of phenotypes spanning from non-progressive haematuria/albuminuria, adult-onset FSGS and classic (severe) disease [4–7]. The most abundant protein in the glomerular based membrane (GBM) is heterotrimeric type IV collagen (α3α4α5) but there exists numerous other proteins that contribute to GBM architecture and turnover. Our own local experience has highlighted a preponderance of undiagnosed Alport syndrome but here we describe an unexplained family with FSGS, where our detailed genetic analysis identified an association with segregating heterozygous variants in *LAMA2* and *LOXL4*, whose encoded proteins serve roles in basement membrane assembly and function.

## Methods

### Patient ascertainment

Patients were recruited at University Health Network, Toronto, ON, Canada after receiving informed consent in accordance with the hospital Research Ethics Board. We obtained longitudinal clinical data and eventually blood, saliva, or isolated DNA. Clinical information was obtained from telephone interviews, questionnaires and physician reports. Genomic DNA was extracted from blood or saliva samples using standard procedures.

### Linkage analysis

Three affected individuals were genotyped with HumanOmni2-5Exome-8-v1-1-A (2,583,651 markers) while 8 samples were genotyped with InfiniumOmni2-5Exome-8v1-3_A1 (2,612,357 markers). Parametric multipoint linkage analysis was performed using Merlin under a fully penetrant dominant model, with a disease allele frequency of 0.0001 [8]. Starting with an original set of 2,482,589 autosomal markers, several marker filtering steps were performed including minor allele frequency (MAF) and linkage disequilibrium (LD) based marker filtering, markers with MAF > 0.4 and those with pairwise r^2^ < 0.1 on each chromosome were kept. This ultimately resulted in a set of 11,335 markers across 22 autosomes which were included in the linkage analyses. Since the affection status of 3 out the 11 samples (7014, 7015 and 7824) was undetermined, linkage analysis was performed on the pedigree 3 times taking into account each of the three possible scenarios – (1) all 3 samples are affected, (2) all are unaffected, and (3) all unknown. Results are shown for scenario 3.

### Exome capture and next-generation sequencing

Whole exome sequencing (WES) was performed by The Centre for Applied Genomics, The Hospital for Sick Children, Toronto, Canada. A shotgun library was made from each sample and captured using the Agilent SureSelect Human All Exon V5 (Santa Clara, CA) according to protocol. The manufacturer’s specifications state that the capture regions total approximately ≈180,000 exons from ≈18,700 genes or 54 Mb. Enriched libraries were then sequenced by 150 bp, paired-end read sequencing on Illumina HiSeq 2500 (Illumina Inc, San Diego, CA).

### *In silico* data processing

Reads were mapped to the hg19 reference sequence using the BWA-backtrack algorithm from BWA v0.5.9 [9]. Duplicate reads were removed using MarkDuplicates from Picard v1.79. Local read realignment around insertions and deletions (indels), base quality score recalibration, and variant calling with UnifiedGenotyper, were accomplished using GATK v1.1–28 [10, 11]. SNP calls were subjected to variant quality score recalibration. Indels were discarded if they overlapped repeat masked regions, and hard-filtered by variant call annotations QualByDepth (QD < 10.0), ReadPosRankSumTest (ReadPosRankSum < -20.0), and Strand Bias (SB > -0.01). Base calling was performed using CASAVA v1.8.2. Copy number variants (CNVs) were identified using XHMM after filtering out regions with extreme GC-content and repeat-masked regions [12, 13].

### Basement membrane measurements

Distances were measured in Fiji/ImageJ using a grid method to obtain a minimum of 100 measurement per individual normalised to the length of the GBM [14]. The total number of measurements for each individual were: 6237: n = 177; 6238: n = 160; 6463: n = 210; 7825: n = 359. The mean ± SEM was calculated and a one-way ANOVA with Tukey’s multiple comparisons test was performed using GraphPad Prism version 8.4.3 for Windows, GraphPad Software, San Diego, California USA, www.graphpad.com.

## Results

**Fig. 1:**
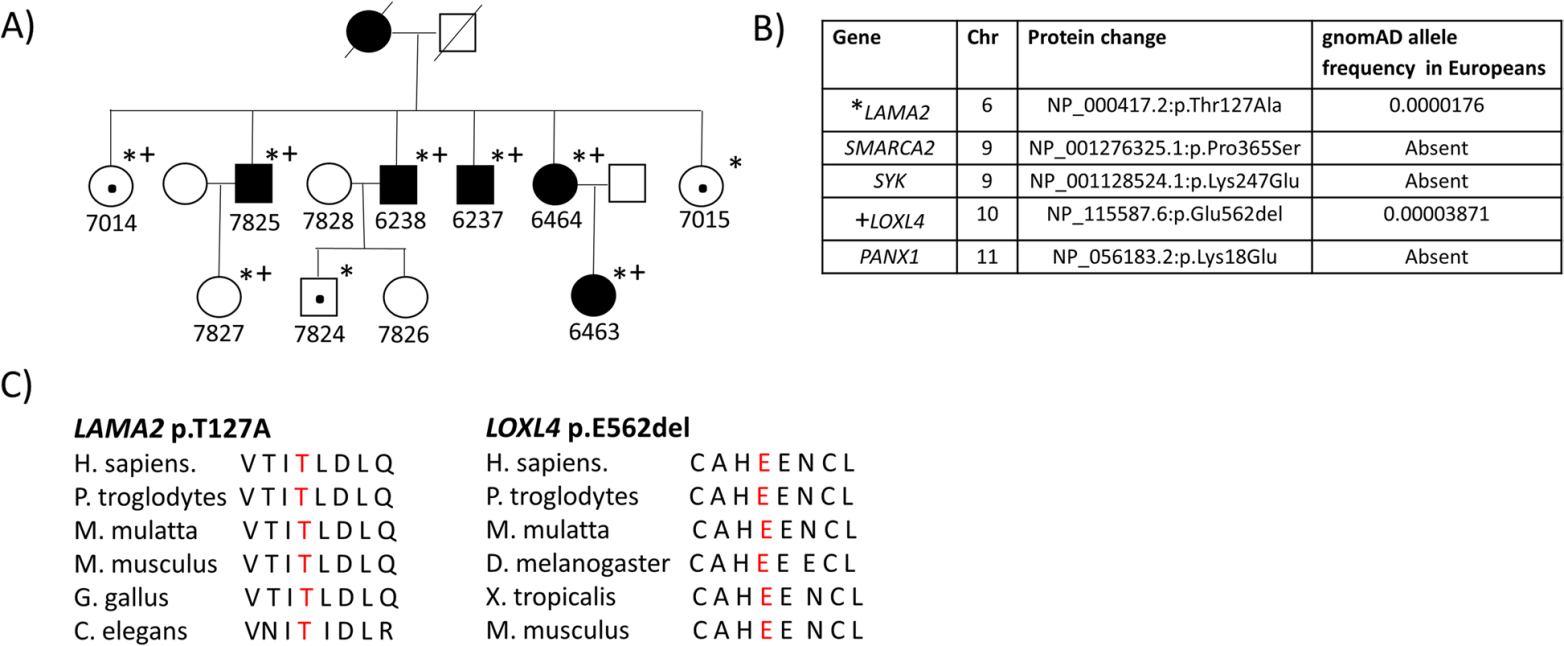
Digenic Inheritance of Rare Variants in *LAMA2* and *LOXL4* in a Family with Autosomal Dominant FSGS. Individuals with dot indicates microalbuminuria and unclear affectation status. **A** Exome Sequencing was performed in 3 affected members (6238, 6237, 6463) of family FSGS 15. **B** Five heterozygous rare variants were identified and sequenced in each relative, with *LAMA2* (*) and *LOXL4* ( +) segregating in affected individuals. **C** These variants affect highly conserved residues across species and are predicted to be deleterious by prediction programs. gnomAD v.2.1.1 accessed May 3, 2020

**Fig. 2:**
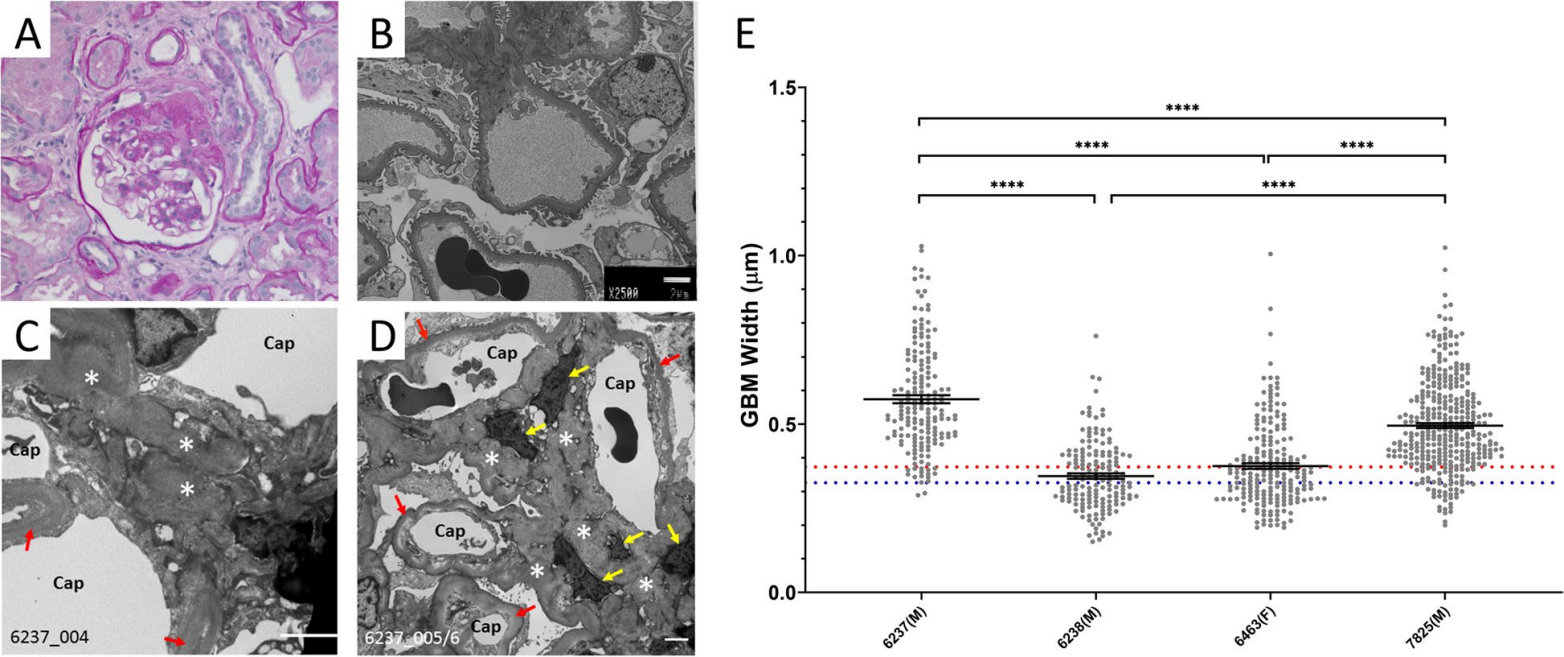
Kidney biopsies from proband, 6238, and sibling 6237. **A** 6238: The first biopsy showed classic segmental sclerosis/hyalinosis lesion on a background of mild mesangial hypercellularity (PAS, 20x). **B** 6238: Ultrastructural examination showed mild podocyte foot process effacement and normal glomerular basement membranes (GBM)(2500x). **C** and **D** 6237: no significant GBM alterations (red arrows) are seen but there are regions of dense extracellular matrix (*), postulated to be mesangial, which appear to enclose cells to the point where only the nucleus is visible (yellow arrows). (E) Comparison of nested averages (mean ± SEM) for each individual: 6237(M): 574.3 nm ± 11.8; 6238(M): 345.7 nm ± 8.1; 6463(F): 375.6 nm ± 8.5; 7825(M): 495.9 nm ± 7.2; all individuals, except 6238(M) & 6463(F), are significantly different from each other (*p* < 0.0001). Dotted lines are the average GBM thickness for males (red; 373 ± 42 nm) and females (blue; 326 ± 45 nm) according to Steffes et al. Lab Invest. 1983 Jul;49(1):82–6

The proband, 6238, presented with proteinuria in his early 20s, with protein excretion rising to up to 8 g/day, and a kidney biopsy at age 41 demonstrated FSGS (Figs. 1 and 2). Additionally, focally duplicated and irregular thickening of the GBM along with focal effacement of podocyte foot processes was observed. His brothers (7825, 6237) were shown to have FSGS in the 4^th^ and 5^th^ decades of life. The proband and his elder brother, 7825, developed end-stage kidney disease (ESKD) in the 5^th^ decade of life while the youngest brother, 6237, has stage 3b A3 CKD at age 59. One sister, 6464, developed proteinuria and impaired kidney function at the time of last follow-up at age 54. Her daughter, 6463, who was found to have FSGS at age 23, had ~ 3.3 g/d of proteinuria and an eGFR of 48 mL/min/1.73m^2^ at age 30. Two of the proband’s sisters, 7014 and 7015, were reported to have proteinuria and no renal biopsies by the 5^th^ decade of life. The proband’s mother was reported to have FSGS, developing ESKD at age 68. The proband’s son, 7824, was described to have proteinuria. 7827 has not had recent screening in his 30s. There was no reported history of hematuria in any of the family members.

Six hundred and twenty-five genes associated with nephropathy, including 59 FSGS, were examined in the family with no segregating rare variants identified [6]. Of note, pathogenic variants causing medullary cystic kidney disease type 1 may lie in a variable-number tandem repeat (VNTR) sequence in *MUC1* and is missed by massively parallel sequencing [15]. However, in this family, the index of suspicion is low for *MUC1*-associated disease given the clinical characteristics consistent with a primary glomerular disorder as evidence by > 3 g/day of protein excretion and supported by pathologic findings rather than a primary tubulointerstitial process [16].

Genotype data from 11 individuals of this European descent pedigree was analyzed for multipoint linkage analysis, which included a set of 11,335 SNPs across autosomes. The segregation of the disease in the pedigree was not compatible with X-linked disease and therefore only autosomal linkage analysis was performed under a fully penetrant dominant model, with the affection status of 5 individuals as affected (6237, 6238, 6463, 6464, 7825), 3 individuals as unaffected (7826, 7827, 7828) and 3 as unknown (7014, 7015, 7824). The maximum LOD score was 0.9028, and it was observed at 388 markers across 11 different chromosomes (chromosome 1, 2, 3, 4, 6, 7, 8, 10, 12, 13 and 18), narrowing the candidate gene(s) to only 3% (388 SNPs/11,335 SNPs) of the genome (Supplementary Fig. 1). Copy number variants (CNV) were also analyzed but there was no co-segregation of any particular CNV in all affected individuals of the family.

Whole exome sequencing was performed in 6237, 6238 and 6463, identifying 5 heterozygous rare variants (minor allele frequency or MAF < 0.01) in the linked regions (Fig. 1). Sanger sequencing of the 5 variants in 11 individuals where DNA was available identified 2 of these to be segregating in all affected individuals, while the other 3 did not (Fig. 1). This included the variants in *LAMA2* (chr6, NM_000426.3:c.380A > G (p.Thr127Ala); MAF 1.76 × 10^–5^) and *LOXL4* (chr10, NM_0002211:c.1684_1686del (p.Glu562del); MAF 3.871 × 10^–5^), which affected amino acid residues that were found to be highly conserved across species (Fig. 1). MAFs were determined by gnomAD v.2.1.1, which contains 125,748 exome sequences and 15,708 whole-genome sequences. Both variants were predicted to be deleterious by in silico programs (Supplementary Tables 1 and 2).

*LAMA2* contributes to laminin networks and localizes to the mesangium while *LOXL4* catalyzes cross linking of collagens and is expressed in glomeruli and tubules. Detailed examination of electron microscopy of basement membranes was undertaken in 4 individuals: 6237, 6238, 6463 and 7825. In 6237, regions of dense mesangial matrix were observed (Fig. 2) but this was not observed in other biopsies of the same individual or the 3 other individuals. The glomerular basement membrane (GBM) thickness was also compared. The average GBM widths (± SEM) were: 574.3 nm ± 11.8 (6237; male), 345.7 nm ± 8.1 (6238; male), 375.6 nm ± 8.5 (7825; male), 495.9 ± 7.2 (all individuals) (Fig. 2). Two of these patients, 6237 and 7825, had wider GBMs than historic control averages (male 373 ± 42 nm, n = 59 male kidney donors; and 326 ± 45 nm, n = 59 female kidney donors) [17].

## Discussion and conclusions

Our comprehensive genetic analysis in this FSGS family consisting of linkage analysis narrowed candidates to 3% (388 SNPs/11,335 SNPs) of the genome. Whole exome sequencing subsequently identified segregating rare variants in *LAMA2* and *LOXL4* as candidate disease genes.

Laminins are found in an intricate lattice of proteins that compose extracellular matrices of organs. *LAMA2* encodes the laminin alpha-2 subunit. In the glomerulus, it heterotrimerizes with laminin beta-1 or 2 (*LAMβ1*, *LAMβ2*) and laminin gamma-1 (*LAMC1*), called laminin 211 or 221, to form the mesangial extracellular matrix [18, 19]. The variant *LAMA2* T127A exists in the laminin N-terminal (LN) domain, which is responsible for trimer-trimer interaction of laminin polymer formation involved in the initiation of basement membrane assembly [20]. Certain variants in *LAMA2* have reported to cause *LAMA2*-muscular dystrophy, an autosomal recessive disorder caused by loss of laminin-211 in skeletal muscle [21]. None of the affected family members had evidence of *LAMA2*-muscular dystrophy. Lama2 protein is also expressed in most tubular segments with the exception of proximal tubules (https://esbl.nhlbi.nih.gov/KTEA/)(22).

*LOXL4* encodes an amine oxidase enzyme that is copper dependent and hypothesized to catalyze the cross linking of collagens and elastins [23]. It is expressed in both glomeruli and tubules (https://www.proteinatlas.org/ENSG00000138131-LOXL4/tissue/kidney; https://gtexportal.org/home/gene/LOXL4; https://esbl.nhlbi.nih.gov/KTEA/)(22). The single amino acid deletion occurs at the C-terminus of the protein, which is highly conserved.

Segregation analysis was performed and deemed not to segregate if not found in an affected individual. However, an unaffected or unknown status individual could have the variant and still satisfy segregation analysis due to reasons of incomplete penetrance or later onset of disease. In this family, ESKD occurs in the 5^th^ to 6^th^ decade of life and some of the female relatives have milder disease.

We designate *LAMA2* and *LOXL4* as candidate FSGS genes due to several study limitations. These include the absence of rigorous functional characterization to support pathogenicity, which is challenging for matrix proteins. Instead we provide renal pathology correlations, demonstrating GBM and mesangial matrix thickening in 3 affected relatives. Alternatively, identification of additional FSGS cases with suspected deleterious variants in *LAMA2* and *LOXL4* will provide more evidence for disease causality. Thus, our report will be of interest to clinicians and genetic groups as sequencing in renal disease becomes more widespread. Though the *LAMA2* and *LOXL4* variants are rare, they are not absent in gnomAD v.2.1.1 but phenotypic data is not available for correlation. However, it is unlikely that these 2 rare variants exist in the same individuals but this is impossible to ascertain based on the summary data that is available in gnomAD. Another limitation is our use of whole exome sequencing, which may not adequately capture some regions and only evaluates coding but not intronic or intergenic sequence.

Based on our findings, we narrow candidates to 3% of the genome and identify coding sequence variants in *LAMA2* and *LOXL4*, which have biologic plausibility, as candidates for disease causation in a family with FSGS. We postulate that the additive effect of digenic inheritance of heterozygous variants in *LAMA2* and *LOXL4* leads to late adult-onset disease in the affected relatives. We further postulate that the absence of clinically significant extra-kidney features including muscular dystrophy is as a result of the impact of the variant (heterozygous missense for *LAMA2,* heterozygous single base pair deletion in *LOXL4*), which should lead to translated protein rather than complete deficiency that can be seen in autosomal recessive disorders like *LAMA2*-muscular dystrophy. Our report will thus be of benefit to the renal community as sequencing in disease becomes more widely applied should more candidate variants in these genes be discovered.

## Supplementary Information

**Additional file 1**. In silico predictions for LAMA2 (Table 1) and LOXL4 (Table 2).

**Additional file 2**. Linkage analysis results including chromosome 6 (LAMA2) and 10 (LOXL4).

## Data Availability

The datasets generated and/or analyzed during the current study are not publicly available but are available from the corresponding author on reasonable request but with restrictions to protect identity of patients.

## Abbreviations

CNV: Copy number variant
ESKD: End-stage kidney disease
FSGS: Focal and segmental glomerulosclerosis
GBM: Glomerular basement membrane
MAF: Minor allele frequency
PAS: Periodic Acid-Schiff
SEM: Standard error of the mean
SNP: Single nucleotide polymorphism
VNTR: Variable-number tandem repeat
WES: Whole exome sequencing
