# Supplementary material for: LAMA2 and LOXL4 are candidate FSGS genes
Source: BMC Nephrol. 2021 Sep 26;22:320. doi: 10.1186/s12882-021-02524-6 (PMC8474709; doi:10.1186/s12882-021-02524-6)
Supplement: Supplementary file 1 — Additional file 1. In silico predictions for LAMA2 (Table 1) and LOXL4 (Table 2). [file 12882_2021_2524_MOESM1_ESM.pdf]

Supplementary Tables 1 and 2

Variant: LAMA2 T127A

Prediction tool	Score	Prediction
SIFT	0.004	Damaging
PROVEAN	-4.34	Deleterious
Mutation Taster	0.999952	Disease causing
Mutation Assessor	4.025	High (predicted functional)
LRT	0.000006	Damaging
PolyPhen2 HDIV	0.91	Probably damaging
PolyPhen2 HVAR	0.68	Probably damaging
FATHMM	-1.25	Tolerated
FATHMM-MLK	0.97836	Damaging
REVEL	0.857	Pathogenic
MetaSVM	0.5744	Damaging
MetaLR	0.6492	Damaging

Variant: LOXL4 E562del

Prediction tool	Score	Prediction	Predicted to cause Nonsense Mediated Decay
SIFT Indel	0.856	Damaging	Yes
PROVEAN	-13.14	Deleterious	-
Mutation Taster	-	Disease causing	-
Mutation Assessor	NA	NA	NA
LRT	NA	NA	NA
PolyPhen2 HDIV	NA	NA	NA
PolyPhen2 HVAR	NA	NA	NA
FATHMM	NA	NA	NA
FATHMM-MLK	NA	NA	NA
REVEL	NA	NA	NA
MetaSVM	NA	NA	NA
MetaLR	NA	NA	NA

NA: score/prediction not available; these programs return data only for missense variants.
