# Supplementary figures and images for: LAMA2 and LOXL4 are candidate FSGS genes
Source: BMC Nephrol. 2021 Sep 26;22:320. doi: 10.1186/s12882-021-02524-6 (PMC8474709; doi:10.1186/s12882-021-02524-6)

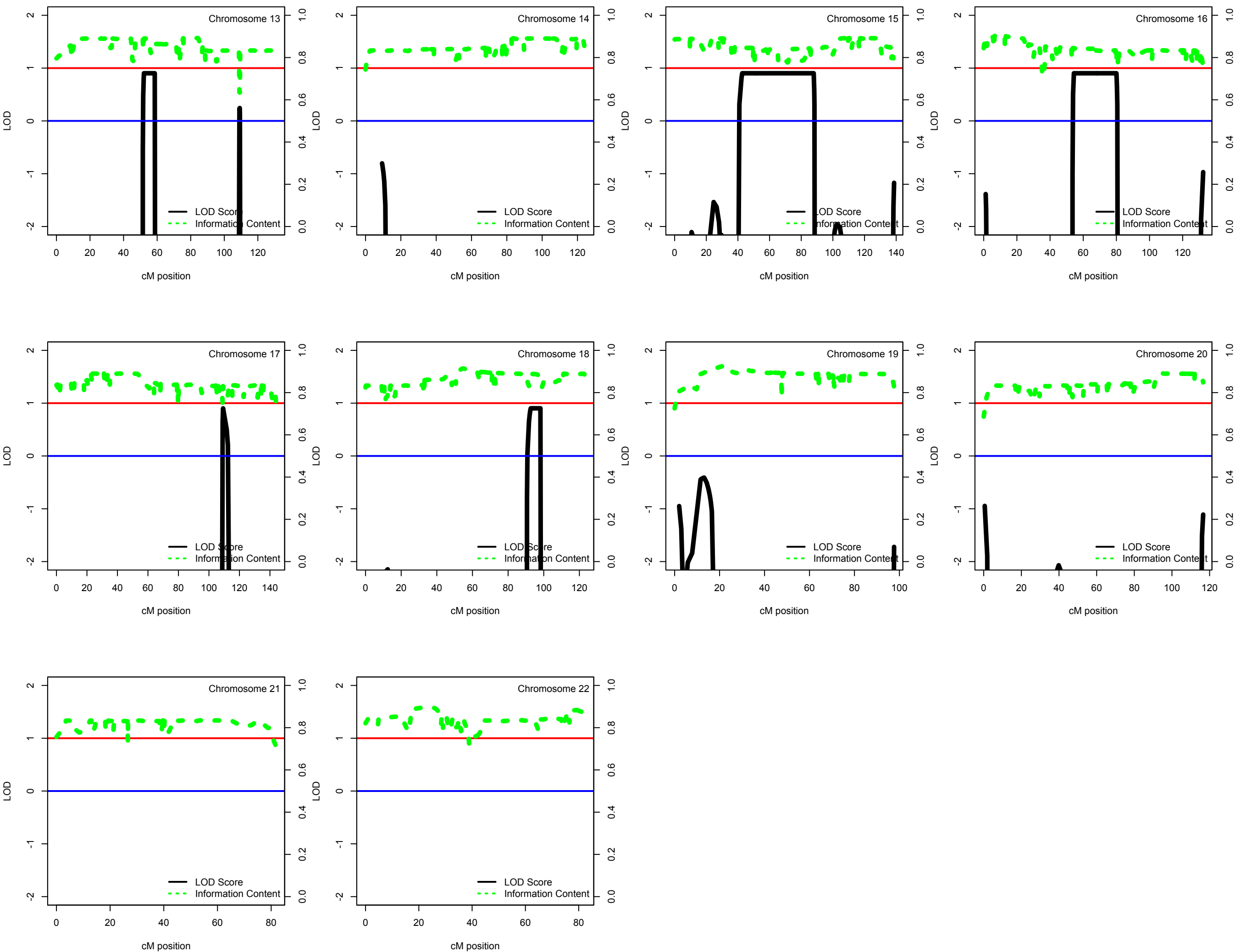

Supplement: Supplementary file 2 — Additional file 2. Linkage analysis results including chromosome 6 (LAMA2) and 10 (LOXL4). [file 12882_2021_2524_MOESM2_ESM.zip › Plot-Barua-FSGS-renal-linkage-ModPed-all3unknw-chr13-chr22R3.pdf]

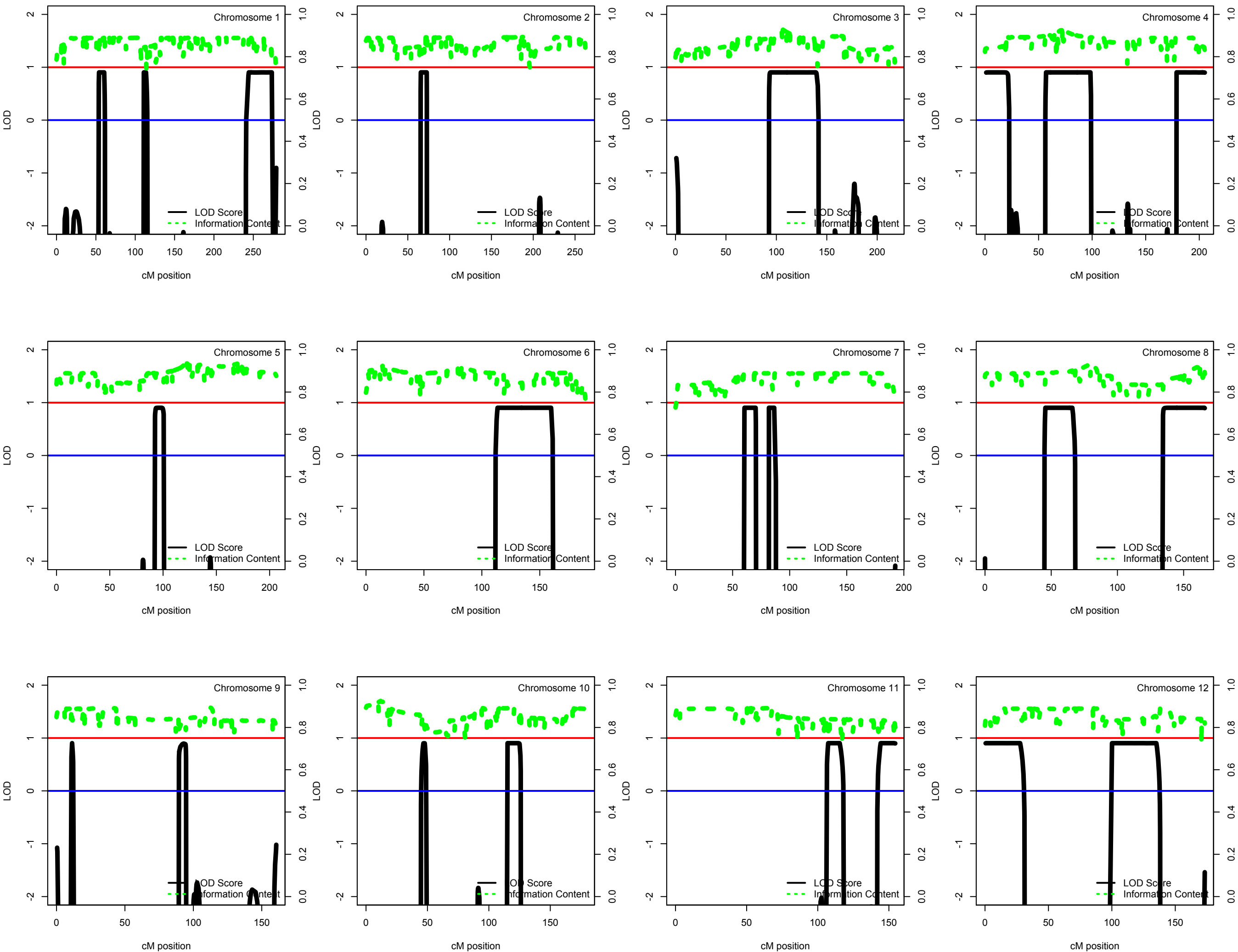

Supplement: Supplementary file 2 — Additional file 2. Linkage analysis results including chromosome 6 (LAMA2) and 10 (LOXL4). [file 12882_2021_2524_MOESM2_ESM.zip › Plot-Barua-FSGS-renal-linkage-ModPed-all3unknw-chr1-chr12R3.pdf]
